# Functional Dissection of Protein Kinases in Sexual Development and Female Receptivity of *Drosophila*

**DOI:** 10.3389/fcell.2022.923171

**Published:** 2022-06-09

**Authors:** Jiangtao Chen, Huan Zhu, Rong Wang, Xiangbin Su, Zongcai Ruan, Yufeng Pan, Qionglin Peng

**Affiliations:** ^1^ The Key Laboratory of Developmental Genes and Human Disease, School of Life Science and Technology, Southeast University, Nanjing, China; ^2^ School of Biological and Medical Engineering, Southeast University, Nanjing, China

**Keywords:** protein kinase, PKA, Akt, CASK, female receptivity, sexual dimorphism, *Drosophila*

## Abstract

Protein phosphorylation is crucial for a variety of biological functions, but how it is involved in sexual development and behavior is rarely known. In this study, we performed a screen of RNA interference targeting 177 protein kinases in *Drosophila* and identified 13 kinases involved in sexual development in one or both sexes. We further identified that PKA and CASK promote female sexual behavior while not affecting female differentiation. Knocking down PKA or CASK in about five pairs of pC1 neurons in the central brain affects the fine projection but not cell number of these pC1 neurons and reduces virgin female receptivity. We also found that PKA and CASK signaling is required acutely during adulthood to promote female sexual behavior. These results reveal candidate kinases required for sexual development and behaviors and provide insights into how kinases would regulate neuronal development and physiology to fine tune the robustness of sexual behaviors.

## Introduction

Protein phosphorylation is an important post-translational modification (PTM). Many enzymes and receptors are activated/deactivated by phosphorylation and dephosphorylation events through kinases and phosphatases (Humphrey, James et al., 2015). Putative orthologous kinase groups have been classified in different species according to the amino acid residue that it phosphorylates, such as STKs (serine/threonine kinases) and TKs (Tyrosine kinases), in which fly and human share several kinases families involved in neurobiology, cell cycle and morphogenesis (Manning, Plowman et al., 2002). In humans, aberrations of kinases have been reported in different types of cancer, as well as neurodegenerative diseases (Ardito, Giuliani et al., 2017; Khan, Kulasiri et al., 2021). Studies in *Drosophila melanogaster* have provided invaluable insights for identifying conserved kinase pathway components and mechanisms that control signaling events involved in development and behaviors (Giese and Mizuno 2013; Mele and Johnson 2019).

Extensive communication between kinases is a prominent feature of signaling networks. For example, Ca^2+^/calmodulin-dependent protein kinase II (CaMKII) is regulated by CASK, a membrane-associated guanylate kinase, in neuronal growth, calcium signaling and learning (Gillespie and Hodge 2013). cAMP-dependent protein kinase (PKA) is a critical kinase involved in several signaling processes in the central nervous system (CNS) (Khan, Kulasiri et al., 2021). The *Drosophila* PKA consists of three catalytic subunits, PKA-C1-3, of which PKA-C1 expressed in the mushroom body (MB) plays important roles in different behaviors, including sleep, learning and memory (Skoulakis, Kalderon et al., 1993; Machado Almeida, Lago Solis et al., 2021). Besides, the loss of PKA-C3 causes copulation defects in male flies (Cassar, Sunderhaus et al., 2018). cAMP binding to the regulatory domain of PKA causes the protein to dissociate, exposing the catalytic subunits, which triggers PKA kinase activity. Both PKA and CaMKII induce cAMP Responsive Element Binding (CREB) activation and enhance specific transcript activity (Borovac, Bosch et al., 2018; Lee, Lin et al., 2018).

The sex determination hierarchy specifies sexually dimorphic aspects of development and behaviors in *Drosophila* (Robinett, Vaughan et al., 2010; Sato and Yamamoto 2014). Female-specific expression of *transformer* (*tra*) along with the non-sex-specific *transformer 2* (*tra2*) control sex-specific splicing of two pivotal downstream genes, *doublesex* (*dsx*) and *fruitless* (*fru*). *dsx* and *fru* are sex-specifically spliced to yield male-specific Dsx^M^ and Fru^M^, and female-specific Dsx^F^ transcription factors (Baker and Ridge 1980; Burtis and Baker 1989; Ito, Fujitani et al., 1996; Ryner, Goodwin et al., 1996). Dsx^M^ and Dsx^F^ are expressed in various tissues and cells, directing most aspects of somatic sex differentiation outside the CNS in both sexes (Lee, Hall et al., 2002; Rideout, Dornan et al., 2010). In the CNS, Fru^M^ is expressed in ∼2000 neurons, controlling sexual behavior in males (Lee, Foss et al., 2000; Manoli, Foss et al., 2005; Stockinger, Kvitsiani et al., 2005; Yu, Kanai et al., 2010), while Dsx^M^ is expressed in ∼900 neurons, most of which co-express Fru^M^. Dsx^M^ regulates courtship intensity as well as sine song production in the presence of Fru^M^ (Villella and Hall 1996; Shirangi, Wong et al., 2016), and the experience-dependent courtship acquisition in the absence of Fru^M^ (Pan and Baker 2014). In females, Dsx^F^ is expressed in ∼700 neurons in the CNS. These *dsx^F^*-expressing neurons, particularly a cluster of pC1 neurons in the central brain, regulate virgin female receptive, post-mating behavior and aggression (Zhou, Pan et al., 2014; Rezaval, Pattnaik et al., 2016; Palavicino-Maggio, Chan et al., 2019; Wang, Wang et al., 2020; Wang, Wang et al., 2021).

Protein phosphorylation controls many cellular processes to coordinate complex functions including sexual development. In *Drosophila*, somatic sexual identity is under the control of a genetic cascade, which requires Hopscotch (Hop), a JAK kinase, to transduce the 2-fold expression differences in a X-linked signal element *unpaired* (*upd*), augmenting the initial sex-determination signal, the expression of *Sex lethal* (*Sxl*) (Avila and Erickson 2007). It has also been found that the kinase Doa (Darkener of Apricot) phosphorylates SR (serine/arginine) proteins, including Tra and Tra2, and is required for proper alternative splicing of *dsx* in both sexes (Du, McGuffin et al., 1998). *Doa* mutant males made fewer copulation attempts and success with both control and *Doa* mutant females (Fumey and Wicker-Thomas 2017). Nevertheless, how different kinases regulate sexual development and behaviors is still rarely known.

To systematically investigate the functions of protein kinases in sexual development and sexual behavior, we screened 177 kinase RNAi lines and identified several kinases, such as Akt and Prpk (p53-related protein kinase, encoded by *Tcs5*), that are crucial in *dsx*-expressing cells for sexual development. We also identified two kinases, PKA and CASK, that regulate virgin female receptivity. We further found that PKA and CASK promote virgin female receptivity by regulating the neuronal projection of ∼5 pairs of pC1 neurons during development and modulating the neuronal physiology of these neurons during adulthood. Our results reveal how specific protein kinases are involved in sexual development and behavior.

## Results

### Identification of Specific Protein Kinases in Sexual Development and Behavior

**FIGURE 1:**
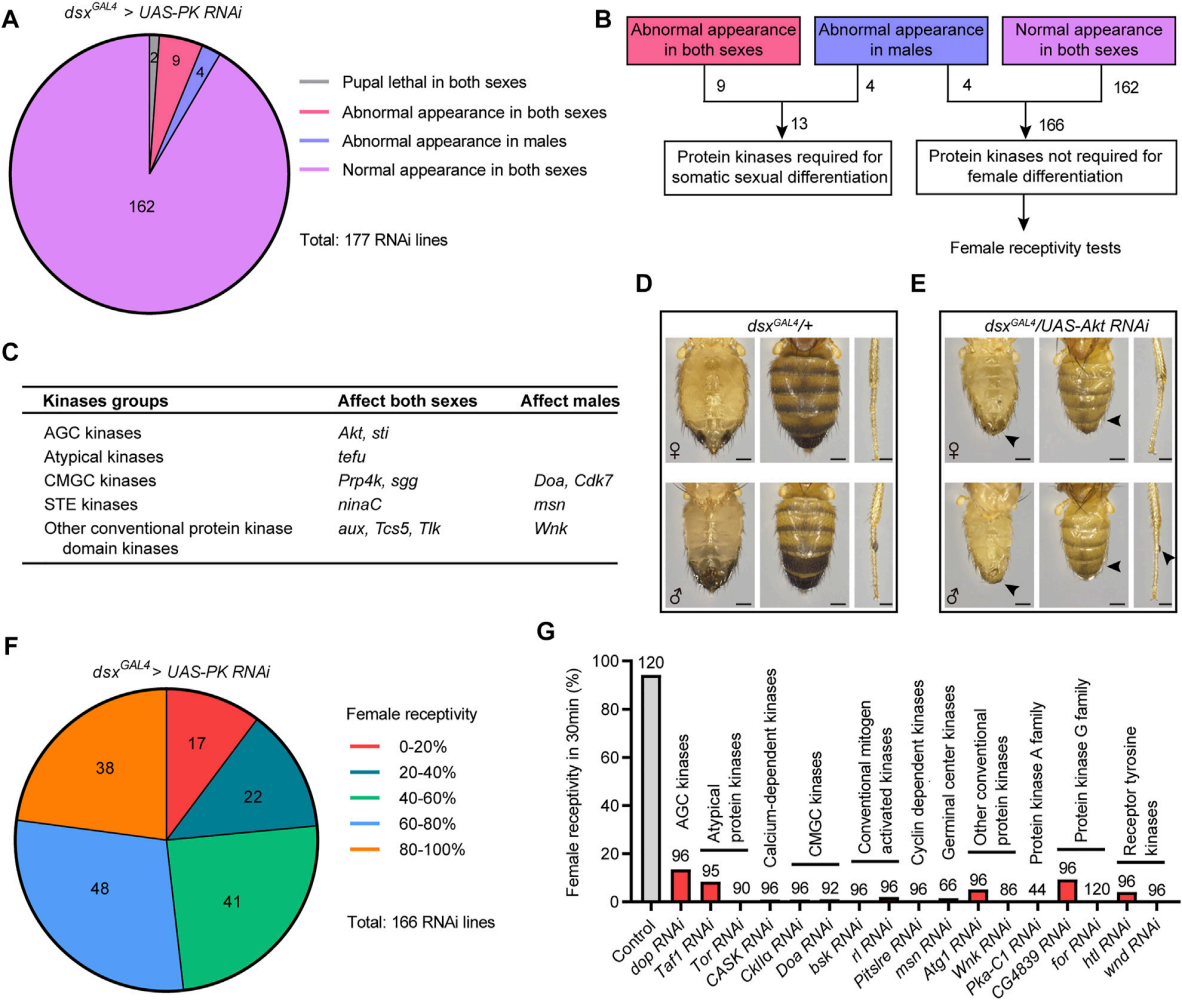
Specific kinases are required for sexual development and female receptivity. **(A)** Statistical results of screening for developmental defects of sex traits. The number in the colored graph represents the number of different RNAi lines targeting protein kinases. **(B)** Protein kinases were divided into two categories, one crucial for somatic sexual development, and the other not required for female differentiation, which were further assayed for virgin female receptivity. **(C)** A summary of protein kinases that are required for somatic sexual development. **(D,E)** Knockdown of *Akt* in *dsx*-expressing cells led to developmental defects in external genitalia, abdominal cuticular pigmentation (scale bar, 0.2 mm) and sex comb (scale bar, 0.1 mm), as indicated by arrowheads. **(F)** Statistical results of screening for unreceptive females. The number in the colored graph represents the number of different RNAi lines targeting protein kinases. **(G)** Knockdown of 17 protein kinases in *dsx*-expressing cells severely reduced female receptivity. The number at the top of the bar indicates the number of tested flies. The genotype of the control group was *dsx^GAL4^/+*; and other genotypes were abbreviations of specific kinase RNAi driven by *dsx^GAL4^*.

To explore the functions of protein kinases in regulating sexual development and sexual behavior, we performed an RNAi screen to knock down the expression of different kinases driven by *dsx^GAL4^* in all *dsx*-expressing cells. We found a small number of kinases (13 out of 177) that were required in *dsx*-expressing cells for sexual development. Among them, 9 kinases were required in both sexes and 4 kinases were required only in males for the development of external sexual morphology (Figures 1A,B). Knocking down most kinases (162 out of 177) in *dsx*-expressing cells did not result in obvious changes in external sexual morphology in either sex (Figures 1A,B).

The kinases required for somatic sexual differentiation belong to different groups of protein kinases, including AGC kinases, atypical kinases, CMGC kinases, STE kinases, and other conventional protein kinase domain kinases (Figure 1C). Knocking down the expression of specific kinase resulted in different intersex phenotypes in flies, often with malformed external genitals and intermedial pigmentation of abdomens (Supplementary Figure S1). For example, when *Akt* was knocked down under the control of *dsx^GAL4^*, flies displayed similar intersex phenotypes between females (*w^−^/yv; dsx^GAL4^/UAS-Akt RNAi*) and males (*w^−^/Y; dsx^GAL4^/UAS-Akt RNAi*), including feminized external genitals and abdominal pigmentations in both sexes, and the significant reduction of sex combs in the forelegs of males (Figures 1D,E). These results suggest that specific kinases are crucial for somatic sexual development.

As above mentioned, knocking down 166 out of 177 protein kinases did not affect somatic sexual differentiation in females, but whether they would regulate female behaviors was unknown. Virgin female receptivity is one of the most important female behaviors that are crucial for reproduction. Thus, we initiated another screen of the 166 protein kinases to identify kinases involved in female receptivity (Figure 1B). We tested virgin receptivity in females with specific protein kinases knocked down in *dsx*-expressing cells over a 30 min period, and calculated the percentage of females successfully mated with males within the test. We found that a large number of kinases were crucial for virgin female receptivity: knocking down 80 kinases reduced female receptivity by ∼40%; in particular, knocking down 17 specific kinases reduced female receptivity by ∼80% (Figures 1F,G and Supplementary Figure S2). Based on the above screening results, we focused on these 17 candidate kinases that did not regulate sexual development but significantly reduced female receptivity after knockdown in *dsx*-expressing cells.

### Identification of Protein Kinases That are Required in the Central Brain for Virgin Female Receptivity

**FIGURE 2:**
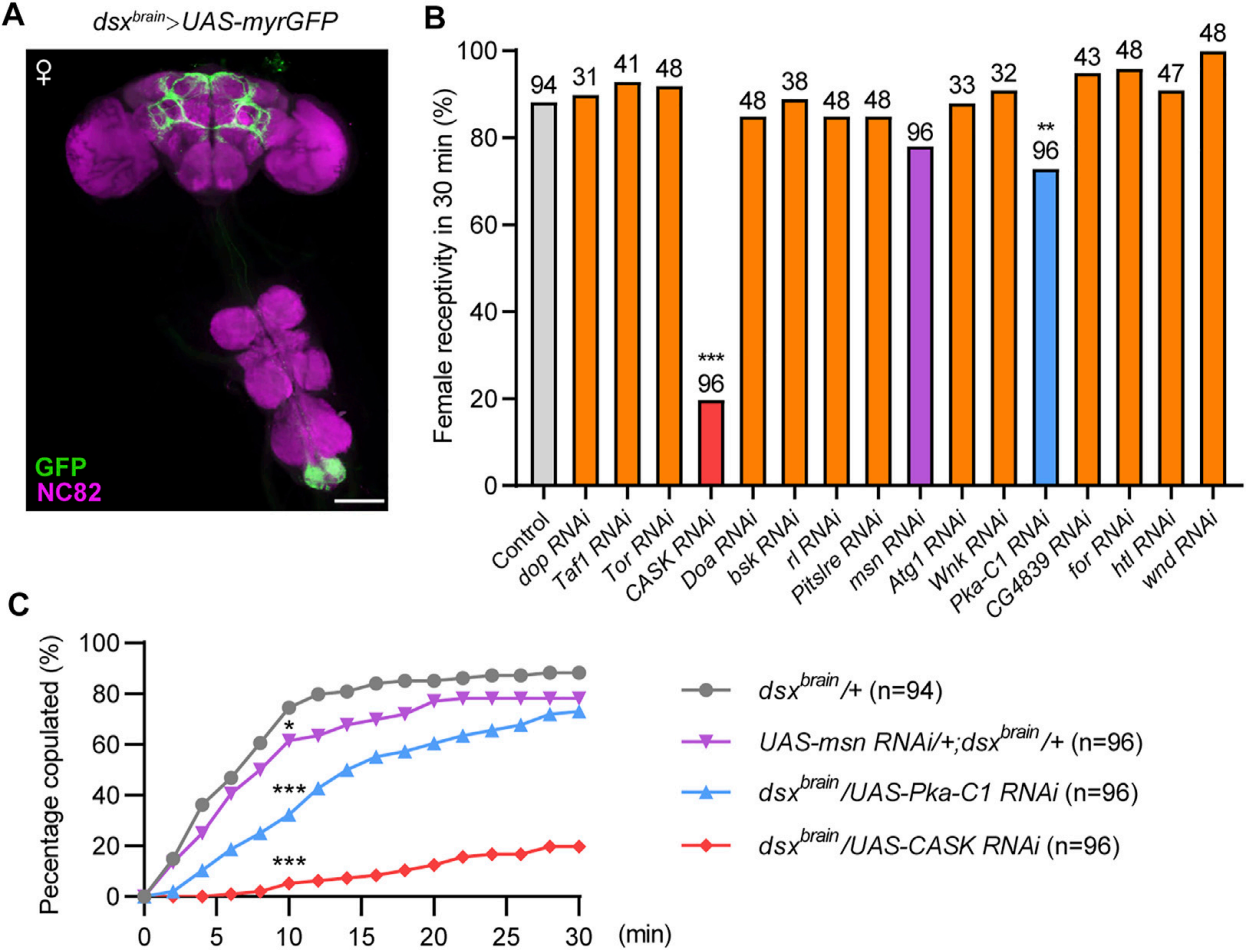
Identification of protein kinases that are required in *dsx^brain^* neurons for virgin female receptivity. **(A)** Expression pattern of *dsx^brain^* in female CNS. Scale bar, 100 μm. **(B)** Compared to the control, female receptivity was significantly decreased with specific kinases knocked down in *dsx^brain^* neurons. The number at the top of the bar indicates the number of tested flies. The control genotype (*dsx^brain^/+*) is *Otd-Flp/+; dsx^GAL4^,tub > GAL80>/+*, and other genotypes were abbreviations of specific kinase RNAi driven by *dsx^brain^*. **(C)** Copulation rates were significantly decreased in virgin females with *PKA*, *CASK* or *msn* knocked down in *dsx^brain^* neurons compared with control females. *p < 0.05 and ***p < 0.001 at 10 min time point, Chi-square test. The number in parentheses indicates the number of tested virgin females paired with wild-type males.

*dsx^GAL4^* is expressed in a variety of tissues with sexually dimorphic features or physiologies, such as the foreleg, cuticle, fat body, reproductive organs, and the CNS. To further investigate the functions of protein kinases in the CNS for female receptivity, we next targeted *dsx* neurons specifically in the brain using *Otd-Flp* expressing FLP specifically in the central brain (*Otd-Flp; dsx^GAL4^,tub > GAL80>*), hereafter referred to as *dsx^brain^* neurons (Figure 2A). We asked whether knocking down the above 17 candidate protein kinases specifically in *dsx^brain^* neurons would affect virgin female receptivity and found that knocking down 14 out of 17 kinases did not affect female receptivity, which suggests that these kinases may regulate female receptivity in *dsx*-expressing cells out of the central brain. Interestingly, knocking down *CASK*, *msn* or *PKA* in *dsx^brain^* neurons significantly reduced female receptivity compared to control female flies (Figures 2B,C), but did not lead to obvious post-mating behaviors, which led us to focus on these three kinases for further study.

### PKA and CASK Signaling in pC1 Neurons is Essential for Virgin Female Receptivity

**FIGURE 3:**
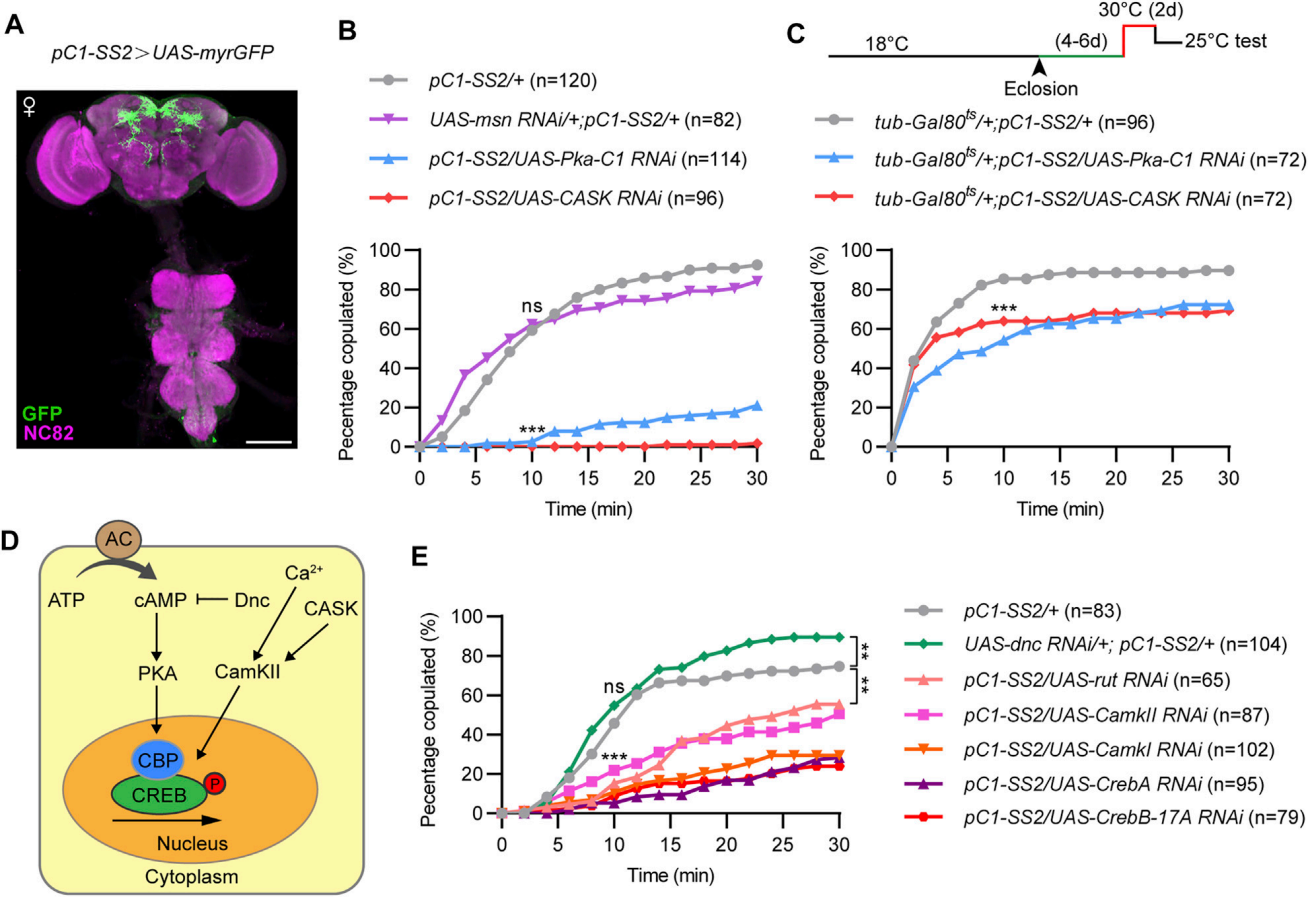
PKA and CASK signaling in pC1 neurons is essential for virgin female receptivity. **(A)** Expression pattern of pC1-SS2 in female CNS. Scale bar, 100 μm. **(B)** Compared to control females, copulation rates were significantly decreased in virgin females with PKA or CASK knocked down in pC1 neurons, whereas copulation rate was not significantly different in msn knockdown females. ns, not significant; ***p < 0.001 at 10 min time point, Chi-square test. **(C)** Knocking down PKA or CASK in 4–6 days old virgin females for 2 days significantly reduced female receptivity. ***p < 0.001 at 10 min time point, Chi-square test. **(D)** Diagram of the PKA and CASK signaling pathway. **(E)** Knockdown of other positive regulators, rather than the negative regulator (Dnc), of PKA and CASK signaling pathway significantly decreased virgin female receptivity. ns, not significant; ***p < 0.001 at 10 min time point. **p < 0.01 at 30 min time point, Chi-square test. The number in parentheses indicates the number of tested virgin females paired with wild-type males.

Previous findings identified a small number of *dsx*-expressing pC1 neurons in the central brain for virgin female receptivity. To further investigate whether the three kinases would function in pC1 neurons for female receptivity, we knocked down *CASK*, *msn* or *PKA* using a previously generated split-GAL4 driver *pC1-SS2*, which specifically labels five pC1 cells (Figure 3A). We found that knockdown of *PKA* or *CASK* in pC1 neurons resulted in a dramatic reduction in female receptivity, while knockdown of *msn* did not affect female receptivity, compared to control females (Figure 3B). The more severe reduction of female receptivity by knocking down *PKA* or *CASK* in pC1 neurons than that in all *dsx^brain^* neurons may be due to a stronger expression in pC1 neurons by the *pC1-SS2* driver.

To further investigate whether *PKA* or *CASK* would be required in pC1 neurons during adulthood for female receptivity, we utilized the temperature sensitive GAL80^ts^ that represses GAL4 activity at 18°C but not at 30°C as we previously used. We found that acutely knocking down *PKA* or *CASK* for 2 days during adulthood significantly reduced female receptivity by ∼20% (Figure 3C). These results indicate that *PKA* and *CASK* play acute roles in pC1 neurons during adulthood for female receptivity. That knocking down *PKA* or *CASK* during adulthood only moderately reduced female receptivity also suggested important roles of these kinases in pC1 neurons during development (see below).

PKA and CASK signaling are involved in the phosphorylation and activation of the transcription factor CREB, which regulates neuronal growth, neuronal differentiation, synaptic plasticity, spatial memory, as well as long-term memory formation. Thus, we tested whether other components of the PKA and CASK signaling pathway are involved in regulating virgin female receptivity, including the cAMP specific adenylyl cyclase (AC) *rutabaga* (*rut*), the cAMP specific phosphdiesterase *dunce* (*dnc*), CamK, and CREB (Figure 3D). As expected, knockdown of the molecules that positively regulate PKA or CASK signaling pathway significantly reduced female receptivity, while knockdown of *dnc*, a negative regulator, did not reduce, but instead slightly increased female receptivity (Figure 3E). Thus, the cAMP-PKA-CREB pathway is crucial in pC1 neurons to regulate female receptivity.

### PKA and CASK Regulate Fine Projection of pC1 Neurons

**FIGURE 4:**
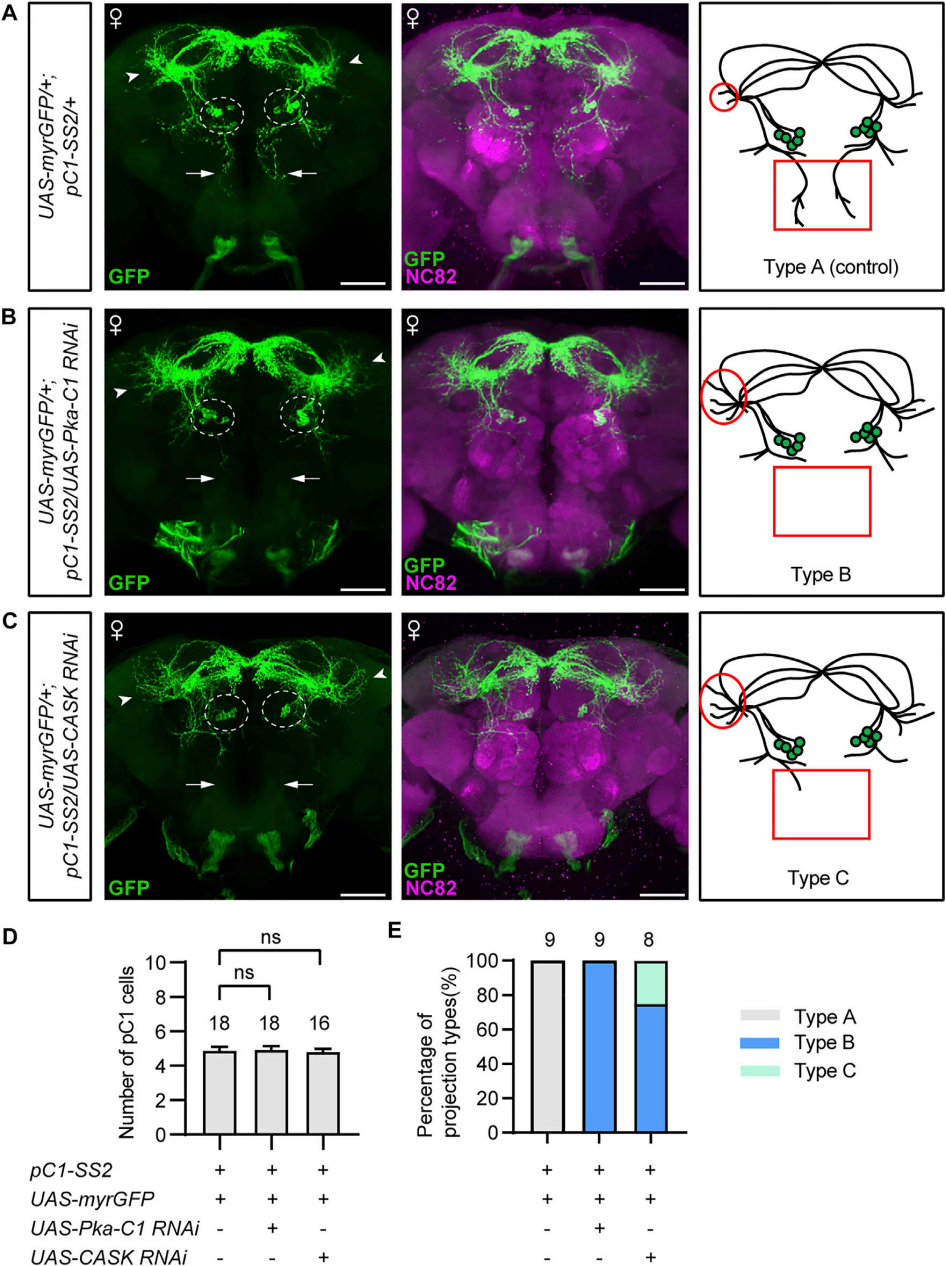
PKA and CASK regulate fine neuronal projection of pC1 neurons. **(A–C)** Morphology of pC1 neurons of different genotypes. Compared with the control **(A)**, pC1 neurons with *PKA* knockdown **(B)** or *CASK* knockdown **(C)** exhibited unchanged number of pC1 cell bodies (dashed circles), loss of the vertical projection (arrows in the left panels, red rectangles in the right panels), and increase of the nerve endings in SMP region (arrowheads in the left panels, red circles in the right panels). Scale bars, 50 μm. **(D)** pC1 cell numbers per hemisphere were not affected by knocking down *PKA* or *CASK*. *n* = 18, 18 and 16, respectively from left to right. ns, not significant, Mann-Whitney U test. Error bars indicate SEM. **(E)** *PKA* or *CASK* knockdown resulted in significant projection defects of pC1 neurons. Type A: regular morphology of pC1 neurons; type B: loss of the vertical projection and increase of the nerve endings in SMP region; type C: weak/unilateral vertical projection and increase of the nerve endings in SMP region. *n* = 9, 9 and 8, respectively from left to right.

The above results indicate that PKA and CASK may function both during development and adulthood in pC1 neurons for female receptivity. To determine whether PKA or CASK regulate the development of pC1 neurons, we examined the morphology of pC1 neurons in female flies with PKA or CASK knocked down. In the control females, there were five pC1 neurons in each hemisphere labeled by the *pC1-SS2* driver, sending projections anterodorsally to the lateral junction of the lateral protocerebral complex and the superior-medial protecerebrum (SMP) (Figure 4A). These pC1 neurons also send projections vertically in the middle of the brain almost to the SOG region (arrows, Figure 4A), probably from the pC1d neurons based on previous studies. We found that pC1 neurons with PKA knockdown displayed abnormal neuronal projection, with the absence of vertical projection (arrows, Figure 4B) and the increase of nerve endings in the SMP region (arrowheads, Figure 4B). Similar deficits were observed in pC1 neurons with *CASK* knockdown (Figure 4C). We suspected that the missing of the vertical neuronal projection might be resulted from the decrease in number of pC1 neurons, e.g., the pC1d neurons, and analyzed the number of pC1 neurons. We found that the number of pC1 cells was still about five in each hemisphere after knocking down *PKA* or *CASK* (Figure 4D). Thus, the loss of the vertical projection in pC1 neuron and the significant increased nerve endings in the SMP region (Figure 4E) were defects of pC1 neuronal projections. These results indicate that PKA and CASK regulate fine neuronal projections of pC1 neurons to promote female receptivity.

**FIGURE 5:**
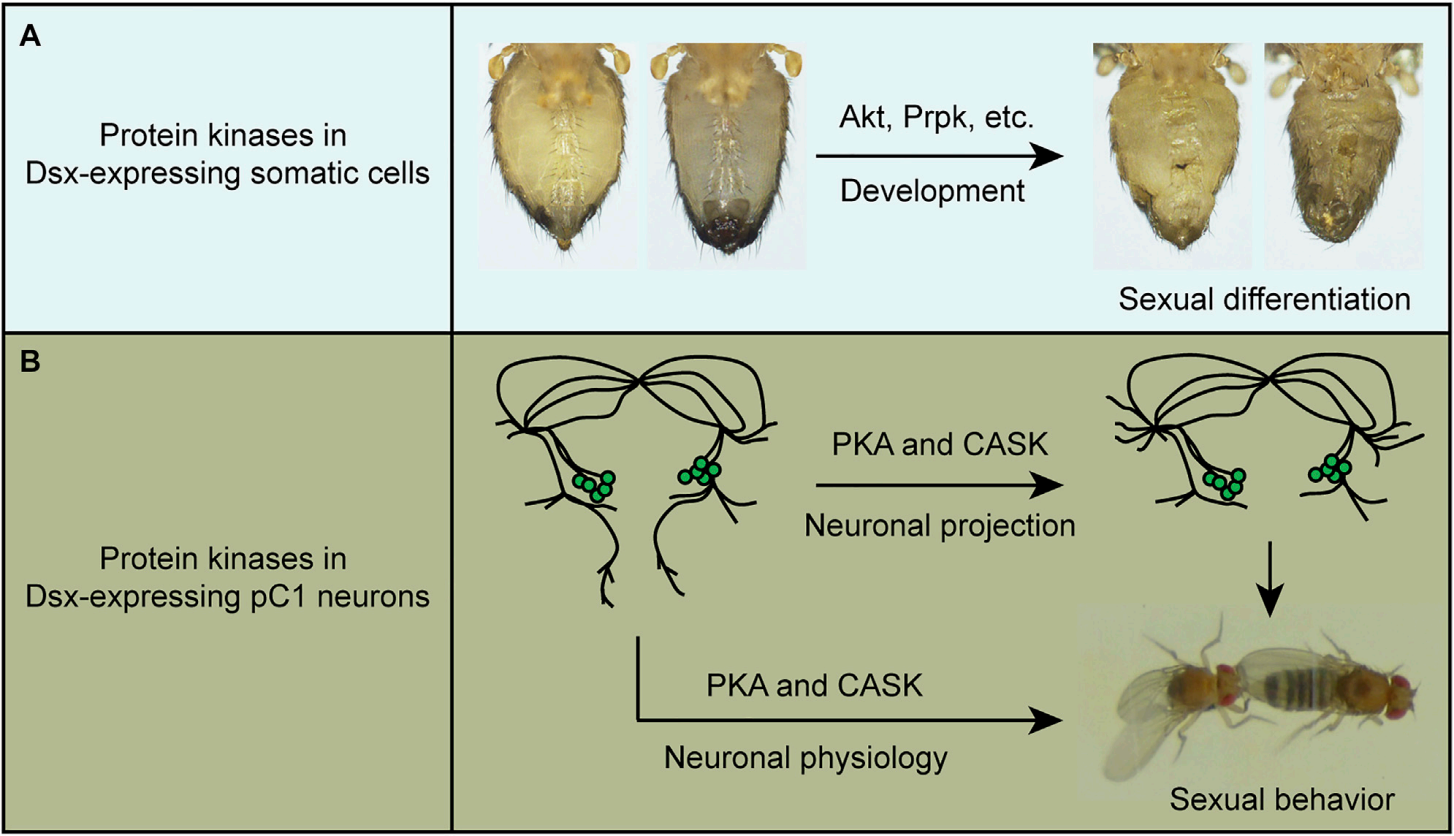
A summary of the functions of protein kinases in sexual development and behavior. **(A)** Protein kinases such as Akt and Prpk are crucial in Dsx-expressing cells for sexual differentiation. **(B)** PKA and CASK regulate both fine neuronal projection of pC1 neurons and their physiology to promote virgin female receptivity.

Taken together, we have identified specific protein kinases that regulate sexual development and virgin female receptivity of *Drosophila*. On the one hand, several kinases, such as Akt, are required in *dsx*-expressing somatic cells to regulate the external sexual morphology (Figure 5A); on the other hand, PKA, and CASK promote receptivity in virgin females by regulating both neuronal projection and physiology of pC1 neurons in the central brain (Figure 5B).

## Discussion

Previous studies have identified several protein kinases involved in sexual development. The JAK kinase Hop regulates expression of the X-linked signal element Upd, which promotes the expression of the sex determination gene *Sxl* (Avila and Erickson 2007). Besides, the kinase Doa phosphorylates Tra and Tra2 to ensure the proper alternative splicing of *dsx* in both sexes (Du, McGuffin et al., 1998). It has been found that hypomorphic *Doa* mutant females, but not males, displayed subtle intersex phenotypes. However, knocking down *Doa* in *dsx*-expressing cells in our study caused a slight abnormality of the external genitals specifically in males. Such differences might be due to the nature of different *Doa* manipulations. In addition, we have identified Akt, Prpk, and a few other kinases required in sexual development. Knockdown of *Akt* not only led to developmental defect of external genitals in both sexes, but also resulted in significant reductions in abdominal pigmentation in both sexes such that the two sexes were difficult to distinguish. The decrease in pigmentation is similar to the phenotypes with perturbating InR and PI3K signaling, which depends on the Akt kinase activity (Shakhmantsir, Massad et al., 2014). Similarly, Prpk transduces the PI3K/TOR signaling to regulate its targets and plays important roles in organ and cell growth (Ibar, Cataldo et al., 2013). In addition to these previously identified pathways, our results also reveal a few other protein kinases that play important roles in the regulation of the sexual development. Among the identified kinases, 4 out of 13 specifically regulate male differentiation. However, we used only one RNAi line for each gene in the screen, and it is possible that the developmental defects were caused by potential off-target of RNAi. Future studies would further test how these kinases regulate sexual development in one or both sexes.

It has been found in rodents that tropomyosin receptor kinase B (TrkB) modulates male sexual motivation (Hawley and Mosura 2019), and the activities of PKA and extracellular signal-regulated kinase (ERK) in distinct brain regions regulate the rapid behavioral shift in male mating reactions (Goto, Nakahara et al., 2015). In *Drosophila*, loss of PKA-C3 or Doa also causes copulation defects in male flies (Fumey and Wicker-Thomas 2017; Cassar, Sunderhaus et al., 2018). However, the role of kinases in female mating behavior is rarely known. We identified that PKA and CASK function in ∼5 pairs of pC1 neurons in the central brain to promote virgin female receptivity. There are at least two categories of function by PKA and CASK. First, we found that PKA and CASK regulate fine projections of pC1 neurons to promote female receptivity. PKA has been found to regulate the fundamental CNS functions including neuronal survival, axonal outgrowth, neuronal development and cognition (Khan, Kulasiri et al., 2021). Our results are generally consistent with these findings regarding to neuronal development. The loss of vertical projection in pC1 neurons in females with PKA or CASK knocked down is likely due to the single pair of pC1d neurons from previous studies on the morphology of single pC1a-e neurons (Wang, Wang et al., 2020). PKA and CASK may specifically modulate the development of pC1d but not other pC1 neurons. Alternatively, PKA and CASK also regulate the development of other pC1 neurons, but the morphological change is trivial and not obviously seen in our study. Second, PKA and CASK also acutely modulate pC1 physiology to promote female receptivity, as knocking down *PKA* or *CASK* for 2 days just before behavioral assays significantly reduced female receptivity. pC1 neurons have been found to regulate several vital functions including virgin female receptivity, egg-laying decision, and aggression (Wang, Wang et al., 2020; Chiu, Hoopfer et al., 2021; Wang, Wang et al., 2021). That PKA and CASK acutely modulate pC1 function provides a molecular pathway that fine tunes the robustness of female sexual and aggressive behaviors.

How kinases function in sexual development and behavior? Regarding to the regulation of sexual development, we speculate that Dsx may be phosphorylated by specific kinases to regulate its function as transcription factor, but the kinases that would phosphorylate Dsx are unknown. *In vitro* phosphorylation experiments would confirm whether Dsx is phosphorylated by specific kinases. Regarding to the regulation of sexual behavior, kinases may function in a general way to affect the formation and activity of neural circuits, like functions of PKA and CASK in pC1 neurons. We speculate that PKA and CASK may act on the transcription factor CREB through different signaling pathways to regulate the neuronal projection and physiology of pC1 neurons. Future studies would testify how the cAMP-PKA-CREB pathway regulates the activity of pC1 neurons, possibly by phosphorylating proteins involved in neurotransmission, to acutely adjust female sexual behaviors.

## Materials and Methods

### Fly Stocks

 Key resources used in this study, such as antibodies and fly stocks, are listed as Table 1. All flies used in this study were raised at 25°C and in 60% humidity with a 12 h light/12 h dark cycle. *dsx^GAL4^* (Rideout, Dornan et al., 2010), *Otd-Flp* (Asahina, Watanabe et al., 2014), *pC1-SS2* (Wang, Wang et al., 2020) and *UAS-myrGFP* (Pfeiffer, Ngo et al., 2010) was used as described previously. *tub-GAL80^ts^* (BDSC_7019) and *tub > GAL80>* (BDSC_38881) were from Bloomington *Drosophila* Stock Center. The *UAS-for RNAi^2dsf07^* (Figures 1G, 2B) was used in the previous study (Peng, Wang et al., 2016). Other RNAi lines used in this study were obtained from the Tsinghua Fly Center at Tsinghua University, with the detailed information listed in the Supplementary Table S1.

**TABLE 1:** Key Resources Table.

Reagent type (species) or resource	Designation	Source or reference	Identifiers	Additional information
Antibody	Rabbit polyclonal anti-GFP	Thermo Fisher Scientific	Cat# A-11122, RRID: AB_221569	IHC (1:1000)
Antibody	Mouse monoclonal anti-bruchpilot antibody (nc82)	Developmental Studies Hybridoma Bank	Cat# nc82, RRID: AB_2314866	IHC (1:50)
Antibody	Donkey polyclonal anti-rabbit, Alexa Fluor 488	Thermo Fisher Scientific	Cat# A-21206, RRID: AB_2535792	IHC (1:500)
Antibody	Donkey polyclonal anti-mouse, Alexa Fluor 555	Thermo Fisher Scientific	Cat# A-31570, RRID: AB_2536180	IHC (1:500)
Chemical compound drug	Normal goat serum (NGS)	Jackson ImmunoResearch Laboratories	Code# 005-000-121 RRID: AB_2336990	
Chemical compound drug	Paraformaldehyde (PFA)	Sigma-Aldrich	CAS# 30525-89-4	4% PFA in PBS
Genetic reagent (*D. melanogaster*)	*dsx^GAL4^*	Rideout, Dornan et al. (2010)	N/A	
Genetic reagent (*D. melanogaster*)	*tub-GAL80^ts^*	Bloomington Drosophila Stock Center	BDSC_7019	
Genetic reagent (*D. melanogaster*)	*tub > GAL80>*	Bloomington Drosophila Stock Center	BDSC_38881	
Genetic reagent (*D. melanogaster*)	*Otd-Flp*	Asahina, Watanabe et al. (2014)	N/A	
Genetic reagent (*D. melanogaster*)	*pC1-SS2*	Wang, Wang et al. (2020)	N/A	
Genetic reagent (*D. melanogaster*)	*UAS-myrGFP*	Pfeiffer, Ngo et al. (2010)	N/A	attP5
Genetic reagent (*D. melanogaster*)	*UAS-Akt RNAi*	Tsinghua University	THU0552	attP2
Genetic reagent (*D. melanogaster*)	*UAS-Pka-C1 RNAi*	Tsinghua University	THU0037	attP2
Genetic reagent (*D. melanogaster*)	*UAS-CASK RNAi*	Tsinghua University	TH02031.N	attP2
Genetic reagent (*D. melanogaster*)	*UAS-msn RNAi*	Tsinghua University	THU4920	attP40
Software, algorithm	ImageJ	National Institutes of Health	https://ImageJ.nih.gov/ij/	
Software, algorithm	Prism 8	GraphPad	https://www.graphpad.com/	

### Female Receptivity Assay

Tester virgin females and 4–6 days old wild-type virgin males were gently aspirated into two layers of the round courtship chambers (diameter: 10 mm; height: 3 mm per layer) and separated by a transparent film between the layers. After about 1 h at 25°C, the film was removed, and behavior was recorded by camera for 30 min. Female receptivity was measured every 2 min as the cumulative percentage of females engaging in copulation within 30 min (Figures 2C, 3B,C,E). Female receptivity at 30 min point was presented in Figures 1G, 2B and Supplementary Figure S2.

### Tissue Imaging

For visualizing the morphological appearances of flies, the wings and legs of 3–5 days old flies with control genotype or specific kinase knocked down were removed under a microscope. The abdomens and the forelegs (Figures 1D,E and Supplementary Figure S1) were captured by a Nikon ShuttlePix P-400R digital microscope.

### Immunostaining and Imaging

Brains of flies were dissected in Schneider’s insect medium (Thermo Fisher Scientific, Waltham, MA) and fixed in 4% paraformaldehyde (PFA) in phosphate-buffered saline (PBS) for 1 h at 4°C. After washing four times in PBST (0.5% Triton X-100 in PBS), Brains were blocked with 3% normal goat serum (NGS) in PBST for 60 min, then incubated with primary antibody diluted in 3% NGS for circa 24 h at 4°C. After washed four times in PBST, Brains were incubated with secondary antibodies diluted in 3% NGS for circa 24 h at 4°C. Primary antibodies used: rabbit polyclonal anti-GFP antibody (1:1000; A-11122, Thermo Fisher Scientific) and mouse monoclonal anti-Bruchpilot antibody (1:50; nc82, DSHB). Secondary antibody used: donkey anti-rabbit IgG conjugated to Alexa 488 (1:500, A21206, Invitrogen) and donkey anti-mouse IgG conjugated to Alexa 555 (1:500, A31570, Invitrogen). Brains were then washed four times in PBST and mounted in Vectorshield (Vector Laboratories H-1000). Stacks of images were obtained by a Zeiss 700 confocal microscope and processed with ImageJ (National Institutes of Health).

### Statistical Analyses

Experimental flies and genetic controls were tested at the same condition. Statistical analysis was performed using GraphPad Prism 8 and indicated inside each figure legend. For female receptivity assay, Chi-square tests were performed to compare two different groups. Mann-Whitney U test was performed for pairwise comparisons of the number of pC1 cells. For the quantification of pC1 projection defects, three different types were defined as type A (regular morphology of pC1 neurons), type B (loss of the vertical projection and increase of the nerve endings in SMP region) and type C (weak/unilateral vertical projection and increase of the nerve endings in SMP region). Type B and C were never observed in control females.

## Data Availability

The original contributions presented in the study are included in the article/Supplementary Material, further inquiries can be directed to the corresponding author.
