# Supplementary material for: Functional Dissection of Protein Kinases in Sexual Development and Female Receptivity of Drosophila
Source: Front Cell Dev Biol. 2022 Jun 9;10:923171. doi: 10.3389/fcell.2022.923171 (PMC9220291; doi:10.3389/fcell.2022.923171)
Supplement: Supplementary file 1 [file DataSheet1.PDF]

Supplementary figures and legends

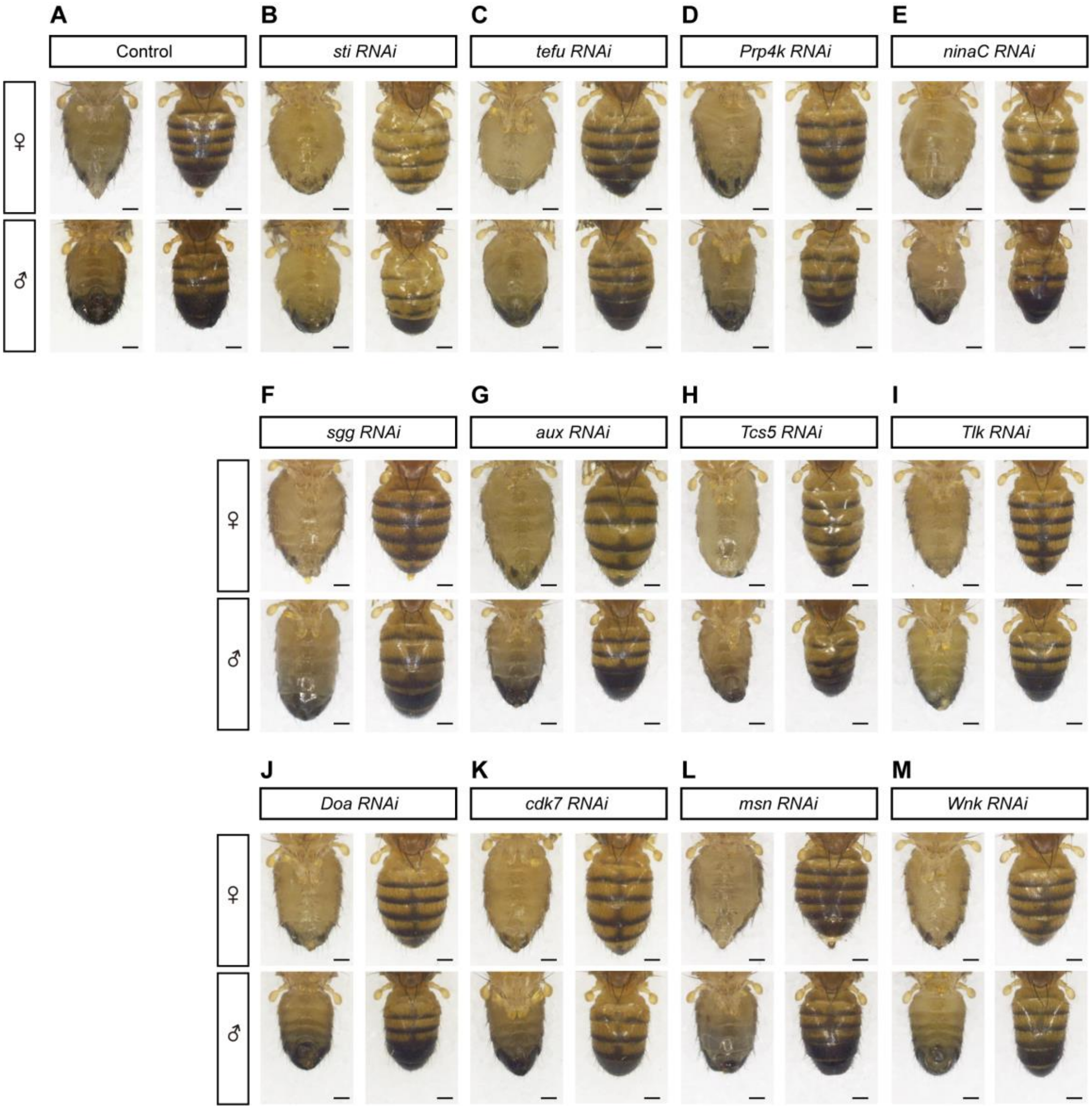

Figure S1. Identification of protein kinases required for somatic sexual development. (A) External sexual traits of control males and females. **(B-M)** Knockdown of specific kinases resulted in different developmental defects in both sexes (B-I) or only in males (J-M). The genotype of the control group was *dsx*^{GAL4}/+; and other genotypes were abbreviations of specific kinase RNAi driven by *dsx*^{GAL4}. scale bars, 0.2mm.

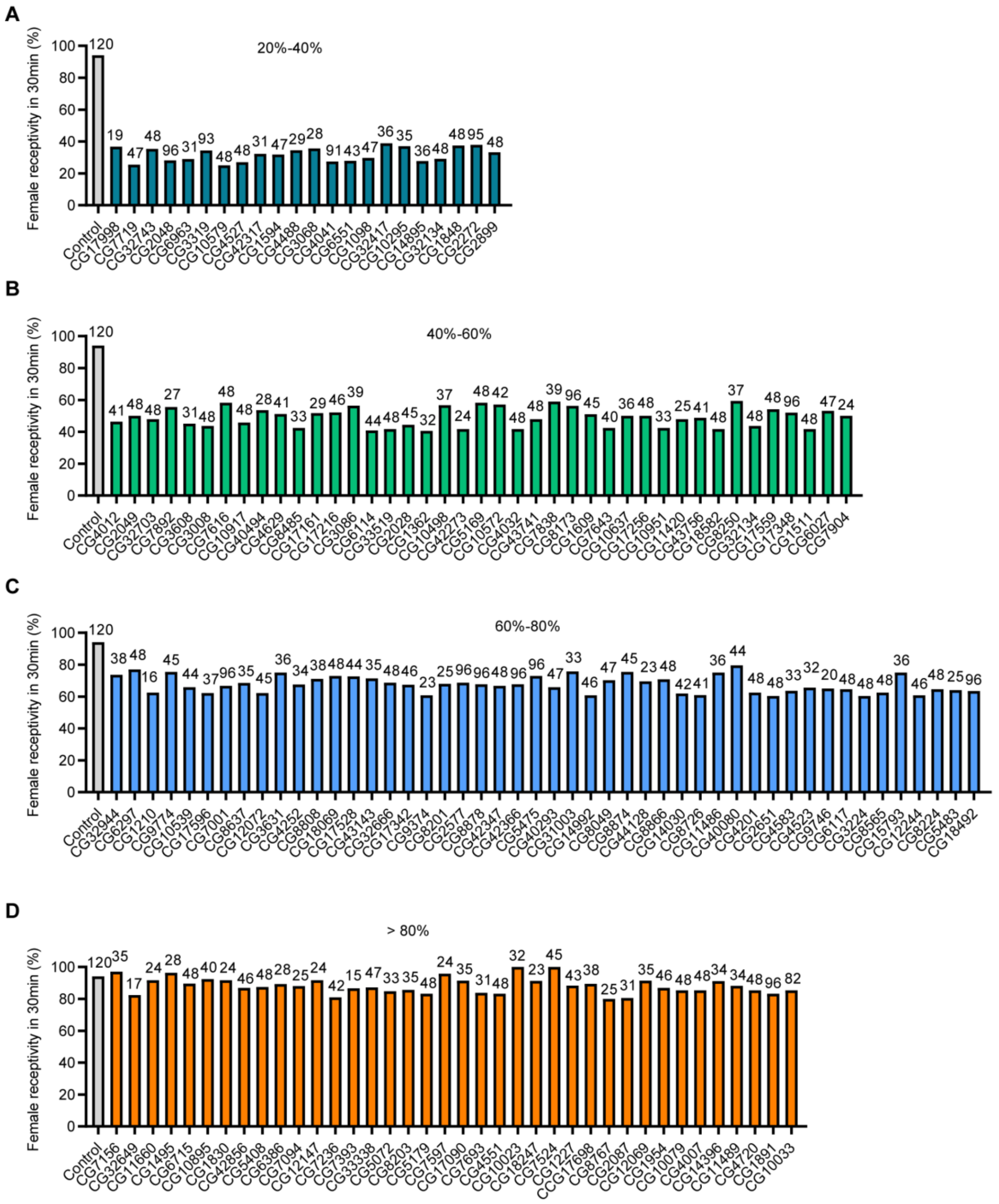

Figure S2. Receptivity of virgin females with different kinases knocked down in *dsx*-expressing cells. (A-D) A list of kinases, knockdown of which resulted in different copulation rates (**A**: 20%-40%; **B**: 40%-60%; **C**: 60%-80%; **D**: >80%). The number at the top of each bar indicates the number of tested virgin females paired with wild-type males. The genotype of the control group was dsx^{GAL4}/+; and other genotypes were abbreviations of specific kinase RNAi driven by dsx^{GAL4}.
